# GuideLiner Balloon Assisted Tracking (GBAT): A New Addition to the Interventional Toolbox

**DOI:** 10.1155/2016/6715630

**Published:** 2016-12-28

**Authors:** Basem Elbarouni, Motaz Moussa, Malek Kass, Olga Toleva, Minh Vo, Amir Ravandi

**Affiliations:** Section of Cardiology, St. Boniface Hospital, University of Manitoba, Winnipeg, MB, Canada

## Abstract

The use of guide extension catheters, such as GuideLiner, allows for increased guide support and facilitates device delivery in tortuous vessels. In cases which the GuideLiner catheter cannot be advanced even with balloon anchoring technique, we inflate a noncompliant balloon protruding from the GuideLiner catheter at nominal pressure and both the GuideLiner and the balloon are advanced over the coronary guidewire through the tortuous segments. This technique can be applied to 5.5 Fr., 6 Fr., and 7 Fr. GuideLiner catheters. This technique is termed GuideLiner Balloon Assisted Tracking (GBAT).

## 1. Introduction

With the advent of drug-eluting stents, an increasing number of difficult lesions are treated by percutaneous intervention (PCI). Despite improvements in-stent profile, stent delivery can still be problematic in complex coronary anatomy. Stent delivery failure occurs in up to 5% of all PCI cases, which can result in suboptimal revascularization and has been associated with increased complication rates [1, 2] The GuideLiner catheter, a mother-and-child catheter that can be delivered deep into the coronary artery, provides excellent support and facilitates stent delivery [3, 4]. To increase success rates, the GuideLiner should be delivered as close as possible to, and sometimes across, the target coronary lesion. This sometimes can be difficult through tortuous and calcified vessels or through previously stented segments. We describe a novel technique that would facilitate delivery of the GuideLiner catheter across difficult segments and increase procedural success rates.

The “GuideLiner Balloon Assisted Tracking” (GBAT) is relatively easy to perform and has a low risk of complications. The main process of GBAT technique involves the following.

**Step 1 .**

**Figure 1:**
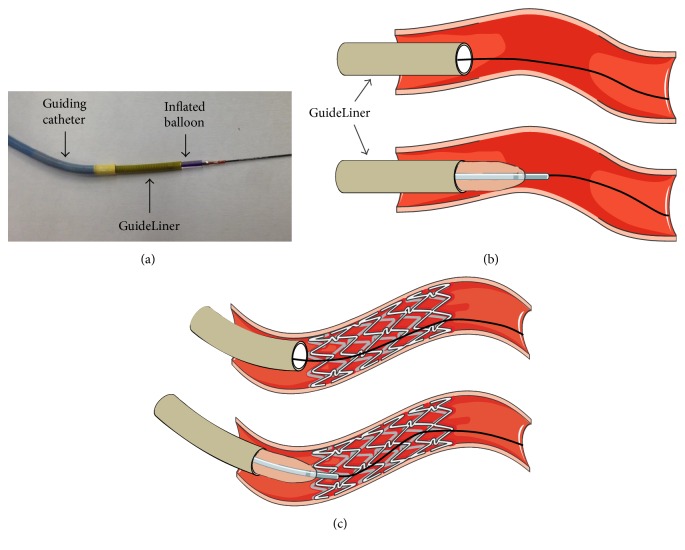
GuideLiner Balloon Assisted Tracking technique. (a) In the image a 6 Fr. GuideLiner is shown with a protruding compliant 2.0 mm × 15 mm balloon. (b) Advancement of the GuideLiner through a tortuous vessel with the compliant balloon at the tip of the GuideLiner. (c) Advancement of the GuideLiner in previously stented segments with the leading balloon tip.

After successful crossing of the lesion with a guidewire and adequate predilatation, a compliant 1.5 × 15 for a 5.5 Fr. GuideLiner or 2.0 × 15 mm balloon for a 6 Fr. GuideLiner is advanced until protruding halfway beyond the tip of the GuideLiner (Figure 1(a)).

**Step 2 .**

The balloon is then inflated to nominal pressure and then both the balloon and the GuideLiner are advanced into the vessel while anchoring the guidewire (Figure 1(b)). Once the GuideLiner is advanced to the target segment the balloon is deflated. The same steps are followed for previously stented segments as shown in Figure 1(c).

## 2. Case  1

**Figure 2:**
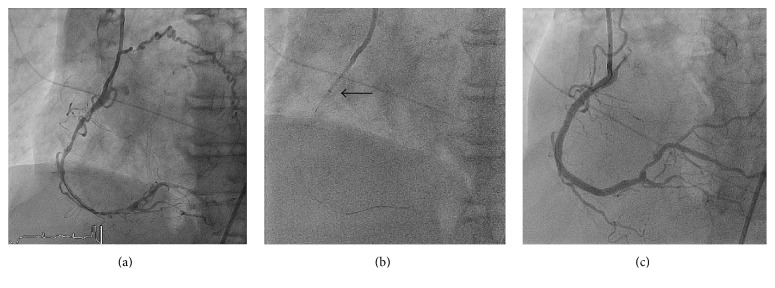
Successful PCI of the RCA using the GBAT technique. (a) Diffusely diseased RCA after predilatation. (b) Advancement of the GuideLiner using GBAT; black arrow indicated the tip of the GuideLiner. (c) Successful PCI of the RCA.

A 72-year-old male presented with a non-ST elevation myocardial infarction (NSTEMI). Coronary angiography showed an occluded mid right coronary artery (RCA) with brisk collaterals from the left anterior descending artery (LAD). The RCA was engaged with a 6 Fr. JR 4 guiding catheter. The lesion was crossed easily with a Pilot 50 wire restoring distal flow. The RCA was diffusely diseased with stenosis extending from the distal RCA to the ostium (Figure 2(a)). Despite predilatation with a 2.0 and a 2.5 noncompliant balloon at high pressure, stent delivery proved quite difficult. A 6 French GuideLiner was used but would not advance beyond the ostium of the RCA. The distal anchoring technique with a 2.5 NC balloon was also attempted but was unsuccessful. Using GBAT technique, a 2.0 × 15 m compliant balloon was inflated protruding halfway out of the GuideLiner at 8 atm. The GuideLiner was easily advanced to the distal RCA (Figure 2(b)) facilitating stent delivery and procedural success (Figure 2(c)). The RCA was stented distal to the ostium with a total of five drug-eluting stents.

## 3. Case  2

**Figure 3:**
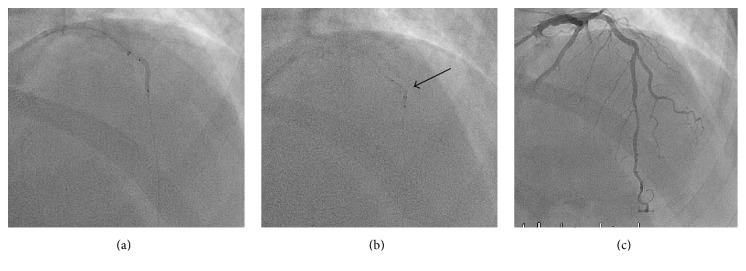
Successful PCI of LAD after using GBAT technique. (a) Predilation of the lesion with a 2.0 × 15 mm noncompliant balloon. (b) Successful advancement of the GuideLiner across the lesion with an inflated balloon at the tip; the black arrow refers to the GuideLiner with the protruding compliant balloon. (c) Successful stent delivery and PCI of the proximal LAD.

A 51-year-old female with CCS III angina and anterior ischemia on MIBI was referred for coronary angiography. PCI was performed on a 90% mid LAD lesion in a tortuous segment (Figure 3(a)). Despite adequate predilatation a stent could not be delivered across the lesion. A GuideLiner was used and was successfully delivered to the proximal edge of the lesion. Even with the added support of the GuideLiner a stent would not cross the lesion. GBAT technique was used to successfully cross the lesion with the GuideLiner (Figure 3(b)). This facilitated stent delivery and procedure completion (Figure 3(c)). The lesion was stented with two contiguous drug-eluting stents (2.25 × 32 Promus Premier DES distally, 2.5 × 24 proximally) with the stented segment postdilated with a 2.5 noncompliant balloon to high pressure.

## 4. Case  3

**Figure 4:**
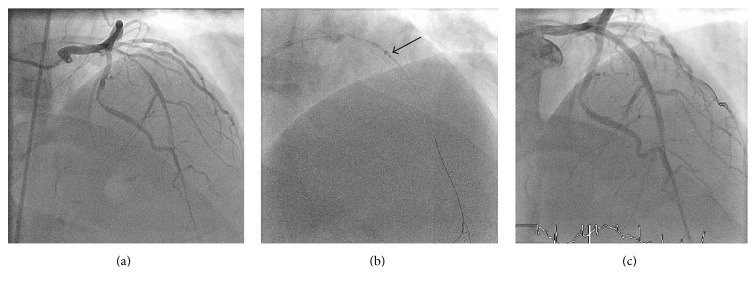
Successful PCI of the LAD using GBAT. (a) Diffusely diseased proximal LAD. (b) GBAT assisted advancement of the GuideLiner passes the proximal stenosis; black arrow indicated the GuideLiner as it passes the lesion with an inflated balloon. (c) Successful PCI to the LAD.

A 63-year-old male presented with STEMI with ST elevation in the anterior leads. Coronary angiography through the femoral approach showed a diffusely diseased proximal LAD with TIMI 2 flow (Figure 4(a)). Next, the left system was engaged with a 6 Fr. XB 3.5 guiding catheter and predilation with a 3.0 × 15 compliant balloon. Even with aggressive predilation there was difficulty in delivering any stents across the lesion. Next, even with the 5.5 Fr. GuideLiner in the proximal LAD we could not deliver a stent across the lesion. We next inflated a 1.5 × 15 mm balloon that was protruding from the GuideLiner to 8 atm and managed to push the GuideLiner and the inflated balloon past the proximal LAD lesion (Figure 4(b)). Next the mid to proximal LAD was stented with DES with great angiographic result (Figure 4(c)).

## 5. Case  4

**Figure 5:**
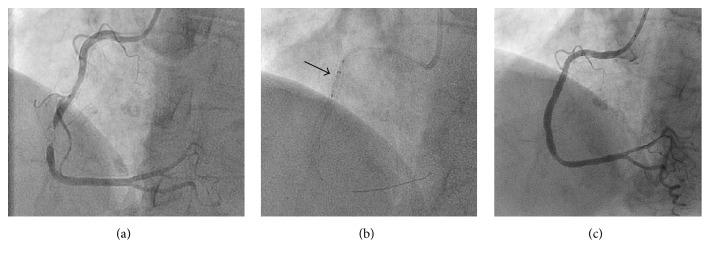
(a) Mid RCA severe lesion with moderate ISR of the proximal vessel. (b) Advancement of the GuideLiner through previously stented segment using GBAT; black arrow indicated the GuideLiner as it passes the lesion with an inflated balloon. (c) Successful postdilation and great angiographic result.

A 72-year-old male presented to hospital with retrosternal chest pain and elevated biomarkers and was referred for inpatient coronary angiography. He has had previous history of CAD with PCI to the proximal to mid RCA with bare metal stents in 2002. The angiogram showed a hazy lesion within the mid RCA as the likely culprit with moderate ISR in the proximal vessel (Figure 5(a)). The RCA was engaged with a 6 Fr. JR4 guiding catheter. There was brisk collateral filling from the RCA. The lesion was crossed with a workhorse wire and predilated with 2.0 compliant balloon. Due to vessel tortuosity we used a GuideLiner to assist in-stent delivery. After stent deployment we could not deliver our 3.5 noncompliant balloon for postdilatation. We also had difficulty advancing the GuideLiner past the proximal stented segment. Next we advanced a 2.0 × 15 mm compliant balloon halfway protruding from the GuideLiner and inflated the balloon at 8 atm (Figure 5(b)). This allowed the GuideLiner to pass the proximal stented segment and allow for postdilation of the stented segmented (Figure 5(c)).

## 6. Discussion

With studies showing excellent outcomes with drug-eluting stents, an increasing number of difficult lesions are treated by PCI. Despite improvements in-stent profile, stent delivery can be challenging in certain lesions. Several techniques are available to operators to help improve success rates in complex PCI procedures. GuideLiner catheter has become a popular and extremely useful tool in treatment of difficult coronary anatomy. It works both by increasing active support and by traversing difficult segments and shortening the distance required to deliver stents. The more distal the GuideLiner is delivered the easier the procedure becomes.

The GBAT technique is a modification of the Balloon Assisted Tracking (BAT) technique, a method described to advance guide catheters through radial arteries with extreme bends and loops [5]. The inflated balloon prevents a “razor effect” of the GuideLiner edge with coronary arty wall or stent struts, allowing easy navigation of curved or tortuous segments and reducing damage to previously stented segments. Balloon assisted deep intubation of guiding catheters has been reported previously to assist with thrombus aspiration [6].

A commonly used technique in GuideLiner delivery is the balloon anchoring technique, in which a balloon is inflated in the target lesion and used as an anchor to facilitate distal delivery of the GuideLiner. This requires the ability to deliver the balloon to a diseased segment of vessel for inflation and carries a high chance of success. In the first case described, the anchor balloon was attempted but was not successful. However, in the second case, we were not able to even deliver a semicompliant balloon distally due to stent interaction; therefore anchor ballooning was not an option. In the third case, as we had to cross the target lesion to deliver a stent, an anchor balloon would have to be inflated distal to the target lesion in normal vessel, potentially causing local trauma that might require stent extension increasing the risk of in-stent restenosis. In the fourth case, the likely reason for difficulty in advancing the GuideLiner was fresh stent struts preventing advancement of the GuideLiner tip as described in Figure 1(c). Therefore, GBAT should be considered a complementary technique to facilitate GuideLiner assisted PCI, especially when anchor ballooning is unfeasible or undesirable, as demonstrated in our cases.

To summarize, GBAT may be considered as a simple and effective approach to facilitate the advancement of the GuideLiner catheter through tortuous, heavily calcified, or previously stented coronary segments. We believe this technique is a useful addition to the armament of interventional skills facilitating high PCI success rates in complex coronary anatomy.
